# Next-generation sequencing guides the treatment of severe community-acquired pneumonia with empiric antimicrobial therapy failure: A propensity-score-matched study

**DOI:** 10.1371/journal.pntd.0012701

**Published:** 2024-12-02

**Authors:** Yuhao Zhao, Yajuan Wang, Yi Li, Chao Song, Pinhua Pan

**Affiliations:** 1 Department of Respiratory Medicine, National Key Clinical Specialty, Branch of National Clinical Research Center for Respiratory Disease, Xiangya Hospital, Central South University, Changsha, China; 2 Center of Respiratory Medicine, Xiangya Hospital, Central South University, Changsha, China; 3 Hunan Engineering Research Center for Intelligent Diagnosis and Treatment of Respiratory Disease, Changsha, China; 4 National Clinical Research Center for Geriatric Disorders, Xiangya Hospital, Changsha, China; 5 Clinical Research Center for Respiratory Diseases in Hunan Province, Changsha, China; 6 Nosocomial Infection Control Center, Xiangya Hospital, Central South University, Changsha, Hunan, China; University of Connecticut, UNITED STATES OF AMERICA

## Abstract

**Background:**

Next-generation sequencing (NGS) is a promising diagnostic tool for pathogens diagnosis. The aim of this study is to evaluate the application of NGS-based antimicrobial therapy on clinical outcomes in severe community-acquired pneumonia (SCAP) patients with empiric antimicrobial therapy failure.

**Methodology:**

We performed a multi-center, retrospective cohort of SCAP patients with initial empiric therapy failure. Propensity score (PS) matching was used to obtain balance among the baseline variables in NGS group (n = 82) and conventional group (n = 82). We compared the diagnostic performance of NGS with conventional microbial culture. We also compared the impact of NGS-based antimicrobial therapy on the prognosis of patients with empirical antimicrobial therapy failure.

**Results:**

The positive rate of NGS was higher than that of conventional pathogen detection methods (92.6% vs. 74.7%, *P* = 0.001). Compared to the conventional group, the NGS group has a considerably higher modifications rate of antibiotic treatment (73.2% vs. 54.9%, P = 0.015). The mortality of NGS group was significantly lower than that of conventional group (28.0% vs. 43.9%, *P* = 0.034). Moreover, the detection of NGS can significantly shorten the ventilation time (*P* = 0.046), and reduce the antibiotic cost (*P* = 0.026).

**Conclusions:**

NGS is a valuable tool for microbial identification of SCAP patient with initial empiric therapy failure.

- Quickly and accurately identifying the pathogen is is crucial for early treatment of severe community-acquired pneumonia, which can significantly improve the efficacy and prognosis. Next-generation sequencing is considered to be a powerful means to identify pathogens of infectious diseases. However, research on severe community-acquired pneumonia, especially on patients with empiric antimicrobial therapy failure, is relatively lacking.
- We found that NGS achieved a microbial etiology in 92.6% of severe community-acquired pneumonia patients with empiric antimicrobial therapy failure. NGS has an obvious advantage in detecting bacteria, viruses, parasites. In addition, NGS was advantageous to identify pneumocystis, adenoviruses, and other rare pathogens.
- Next-generation sequencing was advantageous to identify co-pathogens and mixed infections in severe community-acquired pneumonia patients with empiric antimicrobial therapy failure.
- After adjusting the antimicrobial treatment, the mortality of NGS group was lower than that of conventional group, which indicates that timely NGS detection of pathogens can effectively guide the use of antimicrobial drugs in severe community-acquired pneumonia patients with initial empiric therapy.

## Introduction

Severe community-acquired pneumonia (SCAP) is a major health problem, and the hospital mortality is still high, ranging from 25% to more than 50% [1,2]. The key to early treatment of severe pneumonia is the rapid and accurate determination of pathogens, which is important for efficacy and prognosis [3,4].

Currently, the detection methods for pathogens include morphological detection, microbial culture, nucleic acid amplification, serological pathogen-specific antibody titer detection, and pathogen-specific PCR detection [5–7]. Due to their simple operation, low technical requirements, and certain diagnostic sensitivity and specificity, these methods are currently widely used in clinical practice. However, conventional detection methods have limitations in their sensitivity, specificity, timeliness, and resulting data. Although microbial culture and nucleic acid testing are regarded as the "gold standards" for bacteria and viruses, respectively, the detection time of bacterial/fungal cultures is long, which usually takes days to obtain report, and the positive rate is only 30%-40% [8,9]. The positive rate of PCR detection is high, but specific primers/probes need to be designed, with limited detection types [10]. Serological pathogen-specific antibody titer detection has a window period that cannot be accurately identified [11]. Due to the limitations of conventional detection methods, the causes of approximately 20%-60% of infectious diseases are currently unable to be determined [12].

Next-generation sequencing (NGS) is a high-throughput sequencing technology that objectively identifies all microbial classifications and functional characteristics in a sample [13]. NGS is considered to be a powerful means to identify pathogens of infectious diseases, the application of clinical NGS can theoretically greatly reduce antibiotic abuse and the resistance rate of pathogens [14]. For SCAP patients, the pathogen positivity rate was 90% for NGS versus 39.5% for conventional microbial culture. In addition, NGS can be used to detect mixed pathogens in patients with immunocompromised SCAP and guide personalized antibiotic treatment [15]. However, the high cost of testing, complex steps of analysis and multiple contamination of background microbial have limited its clinical promotion. Research on respiratory infections, especially severe community-acquired pneumonia, is relatively lacking.

This study aims to compare the efficacy and prognosis of NGS-guided drug therapy with conventional detection method-guided therapy in SCAP patients with empiric antimicrobial therapy, and to comprehensively evaluate whether NGS is more advantageous than conventional method detection.

## Materials and methods

### Ethics statement

This study was approved by ethics committee of Xiangya Hospital, Central South University (No.201902017). Written informed consent was provided from each patient or family members at enrolment.

### Study participants

We retrospectively analyzed patients with severe community-acquired pneumonia who admitted to the intensive care unit (ICU) of Xiangya Hospital, People’s Hospital of Hunan Province, Changsha Central Hospital, Second People’s Hospital of Hunan Province, and Xiangtan Central Hospital, Hunan Province, China from April 2019 to November 2020. Diagnosis of SCAP was based on the criteria of the Infectious Disease Society of America/America Thoracic Society (IDSA/ATS) [16].

The inclusion criterion was patients aged between 18 and 85 years who presented with SCAP. The exclusion criteria were as follows: (1) bronchiectasis or diagnosed with active tuberculosis; (2) cannot be excluded for hospital-acquired pneumonia (HAP); (3) emergency observation or hospitalization or mechanical ventilation for more than 48 hours before admission; (4) suffering from severe immunosuppression, multidrug resistance, end-stage renal disease or liver disease (uremia, advanced liver cancer); (5) pregnant. In addition, we excluded patients who improved, died, or withdrew after initial empiric therapy.

**Fig 1:**
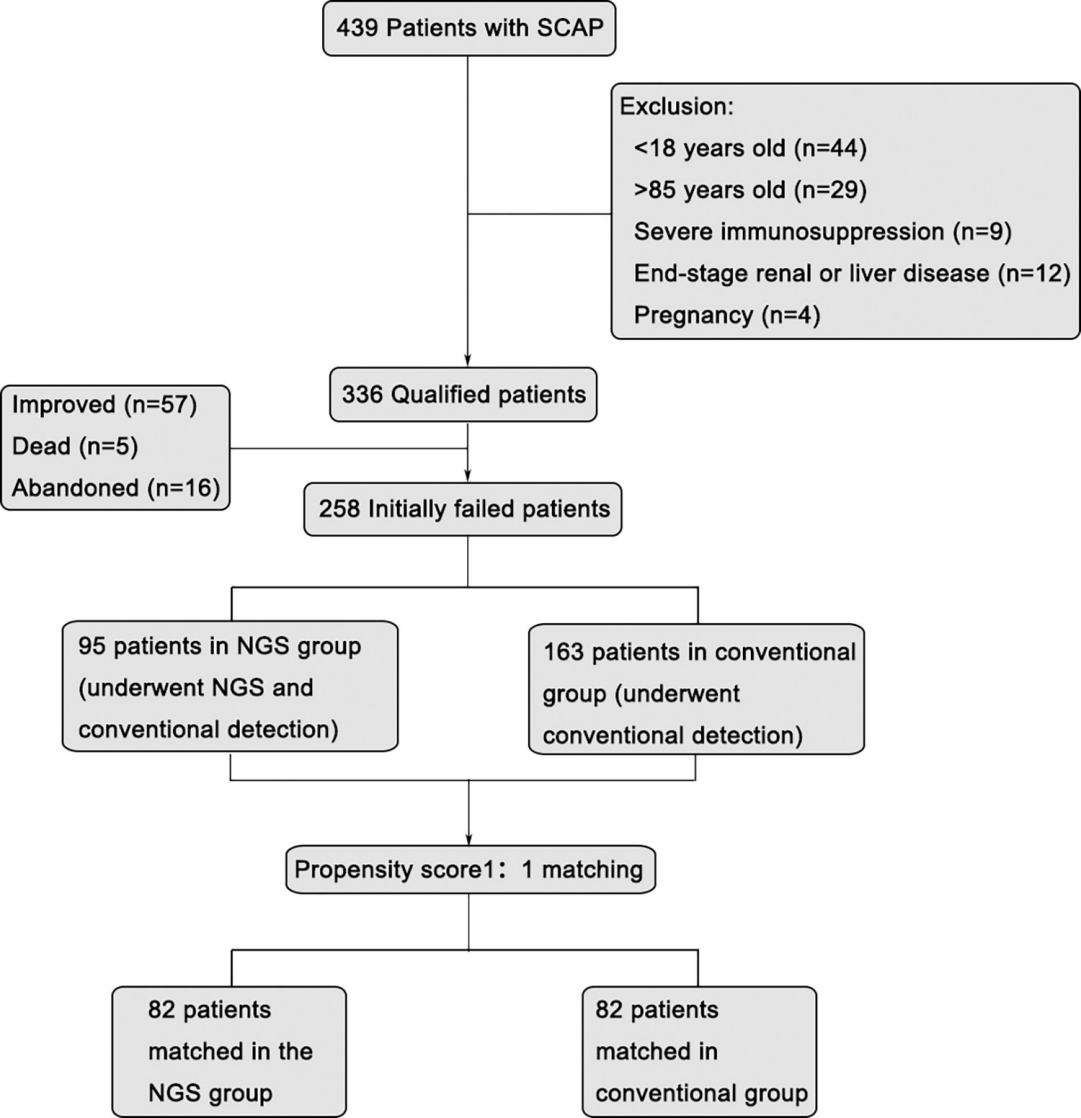
Flow diagram of the sample selection, classification, and comparison.

A total of 439 patients with SCAP were recruited in this study. According to exclusion criteria, patients aged<18 or >85 years (n = 73), with severe immunosuppression (n = 9), end-stage renal disease or liver disease (n = 12), and pregnancy (n = 4) were excluded. Patients who improved (n = 57), died (n = 5), abandoned after initial empiric therapy (n = 16) were also excluded. Ultimately, 258 patients were included in this study. 95 patients underwent NGS detection and conventional detection (NGS group), and 163 patients were only underwent conventional detection (conventional group). After PS matching, 82 patients in NGS group and 82 patients in conventional group were included in our main analyses. Flowchart is provided in **Fig 1**.

### Clinical treatment

Bedside bronchoscopy was performed to obtain bronchoalveolar lavage fluid (BALF) before empirical antimicrobial treatment. The effectiveness of empirical antimicrobial therapy was judged after 72 hours. The definition of effective initial treatment was as follows: body temperature ≤37.2°C, heart rate ≤100 beats/min, respiratory rate ≤24 beats/min, systolic blood pressure ≥90 mmHg, and blood oxygen saturation ≥90% (or PaO2 ≥60 mmHg) [17].

### Bronchoalveolar lavage sample collection

Bronchoscopy was performed by experienced physician. During the surgery, local anesthesia with lidocaine was performed. Briefly, three 20-ml fractions of sterile saline were instilled into the target subsegmental bronchi. The first 20ml was discharged to avoid contamination, whereas the remaining BALF was put into sterile containers and send for NGS test or conventional microbiological tests.

### Construction of DNA library and sequencing

DNA was extracted using the TIANamp Micro DNA kit (Tiangen Biotech) according to standard procedures. DNA fragmentation, terminal repair and PCR amplification were performed using MGIEasy Cell-free DNA Library Preparation Kit (MGI Tech). Agilent 2100 (Agilent Technologies) and Qubit 2.0 (Invitrogen) were used for DNA library quality control. Loaded qualified DNA nanospheres onto the chip, and then performed 20M 50bp single-end sequencing on the MGISEQ-2000 sequencing platform (BGI Genomics). An abundance score of the microbes detected by NGS from each patient was calculated as the sum of the log (DNA-Seq) and log (RNA-Seq) reads per million alignments at the genus level according to our previous research [15,18]. The high-scoring microbes were considered significant microbes.

### Bioinformatic analysis

Bioinformatics analysis was performed on samples with raw data (>10 million). The human host sequence mapped to the human reference genome (hg19) using the Burrows-Wheeler alignment tool (v0.7.17 with default parameters). Microbial classification was performed by mapping the sequencing reads to a reference microbial database that includes the genomes of archaea, bacteria, fungi, protozoa, viruses, and parasites selected from the NCBI genome database [19].

### Data collection and outcome measures

Routine microbiological tests were performed on all patients, including culture (fungal and bacterial), PCR (influenza virus, adenovirus, coronaviruses, syncytial virus, parainfluenza virus, and enterovirus), and serological test (influenza A and B viruses, Legionella pneumophila, Chlamydia pneumoniae, and Mycoplasma pneumonia). In NGS group, NGS and routine microbiological tests were performed. The primary outcomes of this study were mortality and rate of improvement (Improvement means that the patient’s symptoms and signs have improved after treatment, but have not yet reached the cure standard).

### Statistical analysis

Propensity score (PS) matching was used to obtain balance between the NGS and conventional group [20–23]. Propensity scores were calculated using a multivariate logistic regression model with ropensity scores were calculated using a multivariate logistic regression model with several potential confounding factors, namely, sex, age, chronic heart failure, diabetes, chronic liver disease, chronic obstructive pulmonary disease (COPD), cerebrovascular disease, tumor, sepsis, acute respiratory distress syndrome (ARDS), multiple organ dysfunction syndrome (MODS), PaO2/ FiO2, SOFA score, APACHE II score, and CURB-65 score. The NGS group was matched to the conventional group on a 1:1 basis according to the nearest-neighbor method. The continuous variables were expressed as means ± standard deviations (SDs) or medians ± interquartile ranges (IQRs) where appropriate. The matching tolerance was controlled at 0–1, the final match tolerance was 0.05, and the difference was selected according to the actual situation.

## Results

### Patient characteristics

After PS matching, 82 patients in the conventional group and 82 patients in the NGS group were analyzed. The patient clinical baseline characteristics were presented in **Table 1**. There were no significant differences in sex, age, PaO2/FiO2, comorbidities (chronic heart failure, diabetes, chronic liver disease, COPD, cerebrovascular disease, or tumor), complications (sepsis, MODS, ARDS), PCT, D-dimer, SOFA score, or APACHE II score between two groups. The demographics and baseline characteristics of patients without PS matching were presented in **S1 Table**.

**Table 1:** Patient clinical baseline characteristics after PS matching.

Parameter	NGS group (n = 82)	conventional group (n = 82)	P value
Age, year	57.7 (14.3)	58.9 (14.3)	0.59
Sex, male/female	61/21	61/21	1.00
**Comorbidities, n (%)**			
chronic heart failure	21 (25.6)	24 (29.3)	0.60
diabetes	11 (13.4)	10 (12.2)	0.82
chronic liver disease	20 (24.4)	20 (24.4)	1.00
COPD	10 (12.2)	15 (18.3)	0.28
cerebrovascular disease	33 (40.2)	31 (37.8)	0.75
tumor	10 (12.2)	13 (15.9)	0.50
**Complications, n (%)**			
sepsis	22 (26.8)	19 (23.2)	0.59
MODS	12 (14.6)	8 (9.8)	0.34
ARDS	14 (17.1)	13 (15.9)	0.83
PCT(μg/L)	0.7(0.2–3.8)	0.7 (0.2–3.3)	0.86
D-dimer	1.7 (0.7–3.2)	1.5 (0.9–2.9)	0.90
PaO2/ FiO2(mmHg)	187.6 (104.0)	180.7 (87.8)	0.65
SOFA score	4 (3–6.25)	4 (3–7)	0.66
APACHE II score	16.0 (7.1)	16.2 (6.0)	0.90
NE%	81.7 (14.2)	83.4 (15.3)	0.49
PLT (10^9^/L)	187.0 (120.9)	204.0 (132.4)	0.40
ESR (mm/h)	72.0 (34.0–96.0)	71.5 (40.8–111.0)	0.38
CRP (mg/L)	126.69 (87.2)	104.94 (70.5)	0.11

COPD: chronic obstructive pulmonary disease; MODS: multiple organ dysfunction syndrome; ARDS: acute respiratory distress syndrome; PCT: procalcitonin; PaO2 /FiO2: ratio of arterial oxygen partial pressure to fractional inspired oxygen; SOFA score: Sequential Organ Failure Assessment score; APACHE II: Acute Physiology and Chronic Health Evaluation; NE%: percentage of neutrophils; PLT: platelets; ESR: erythrocyte sedimentation rate; CRP: C-reactive protein; SD: standard deviation; IQR: interquartile range.

### Comparison of outcomes between NGS and conventional groups

**Table 2** showed that the mortality of NGS group was lower than that of conventional group (28.0% vs. 43.9%, *P* = 0.034). After adjusting the antimicrobial treatment, the improvement rate of NGS group was significantly higher than that of conventional group (65.9% vs. 35.4%, *P* = 0.001). There were significant differences in the mechanical ventilation time (142 [30–245] vs. 174 [67–332], *P* = 0.046), antibiotic cost (22198.9 vs. 26894.7, *P* = 0.026), and antibiotic cost/day (821.9 [568.4–2001.5] vs. 1283.4 [838.0–2206.2], *P* = 0.043). However, there was no significant difference in the length of hospital stay (*P* = 0.420). The primary outcomes of patients without PS matching were presented in **S2 Table**.

**Table 2:** Clinical Outcomes of the NGS group and conventional group.

Parameter	NGS group (n = 82)	Conventional group (n = 82)	P Value
**Primary outcomes**			
Mortality during hospitalization (%)	23/82 (28.0)	36/82 (43.9)	0.034
Improvement rate during hospitalization (%)	54/82 (65.9)	29/82 (35.4)	0.001
**Second outcomes**			
**Duration of mechanical ventilation**			
Mechanical ventilation time	142 (30–245)	174 (67–332)	0.046
Ventilator-free hours	216 (9–336)	120 (4–360)	0.180
**Length of hospital stay(day)**			
ICU hospitalization time	10 (6–16)	10 (5–18)	0.650
Total length of hospital stay	17 (11–23)	16 (10–22)	0.420
**Expenses of antibiotic (RMB, Yuan)**			
Antibiotic cost	22198.9 (14589.6)	26894.7 (24370.8)	0.026
Antibiotic cost/day	821.9 (568.4–2001.5)	1283.4 (838.0–2206.2)	0.043

ICU: intensive care unit

### Comparison the change of clinical indicators from admission to discharge

**Fig 2:**
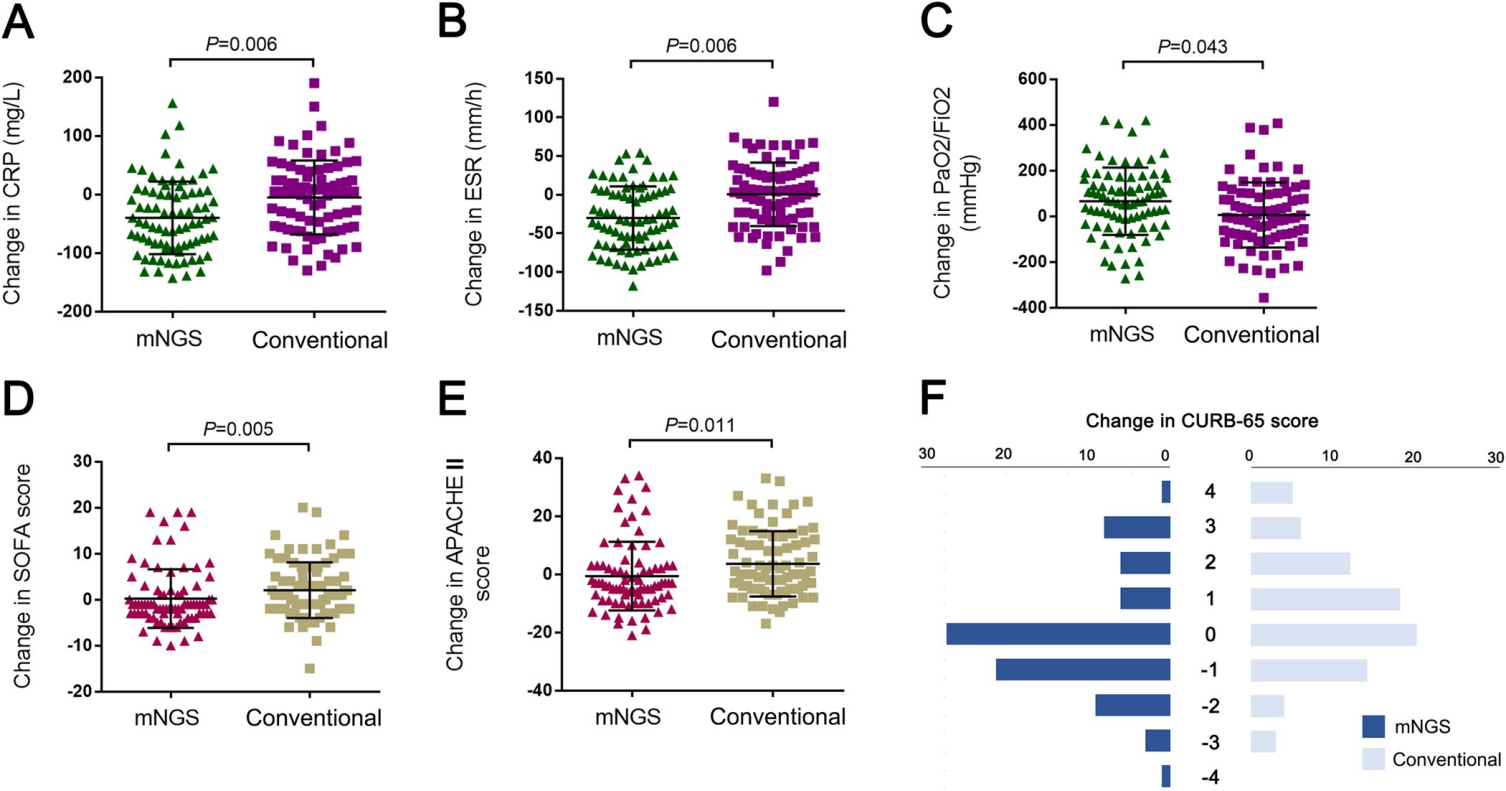
Comparison of clinical indicators change between NGS and conventional groups. (A) change in CRP, (B) change in ESR, (C) change in PaO2/FiO2, (D) change in SOFA score, (E) change in APACHE II score, and (F) change in CURB-65 score. P-values indicate the differences between patients in the NGS and conventional group. CRP, C-reactive protein; ESR, erythrocyte sedimentation rate; PaO2 /FiO2, ratio of arterial oxygen partial pressure to fractional inspired oxygen; SOFA score, Sequential Organ Failure Assessment score; APACHE II, Acute Physiology and Chronic Health Evaluation.

We analyzed the change of clinical indicators from admission to discharge between the NGS and conventional groups (**S3 Table**). There were significant differences in the change in ESR (-31.0 [-55.0- -2.0] vs. 0.5 [-17.2–30.0], *P* = 0.006), change in CRP (-63.0 [-113.2–0.0] vs. 3.0 [-60.3–39.4], *P* = 0.006), change in NT-proBNP (4.3 [-1839.0–2748.0] vs. 898.0 [-404.0–6172.0], *P* = 0.005), change in PaO2/ FiO2 (66.9 [-43.1–170.3] vs. 6.5 [-82.4–113.1], *P* = 0.043), change in SOFA score (-1 [–3–2] vs. 1 [-2-6.5], *P* = 0.005), change in CURB-65 (0 [–1–1] vs. 0.5 [–1–2], *P* = 0.002) and change in APACHE II (-3 [–8–3] vs. 0 [–5–12], *P* = 0.011) (**Fig 2**). However, there were no differences between the two groups in terms of change in percentage of neutrophils, change in percentage of lymphocyte, change in platelets, change in procalcitonin, change in D-dimer, change in aspartate aminotransferase, change in alanine aminotransferase, change in urea, change in creatinine. The change of clinical indicators from admission to discharge without PS matching were presented in **S1 Fig.**

### Comparison of diagnostic performance between NGS and conventional microbiological test

**Fig 3:**
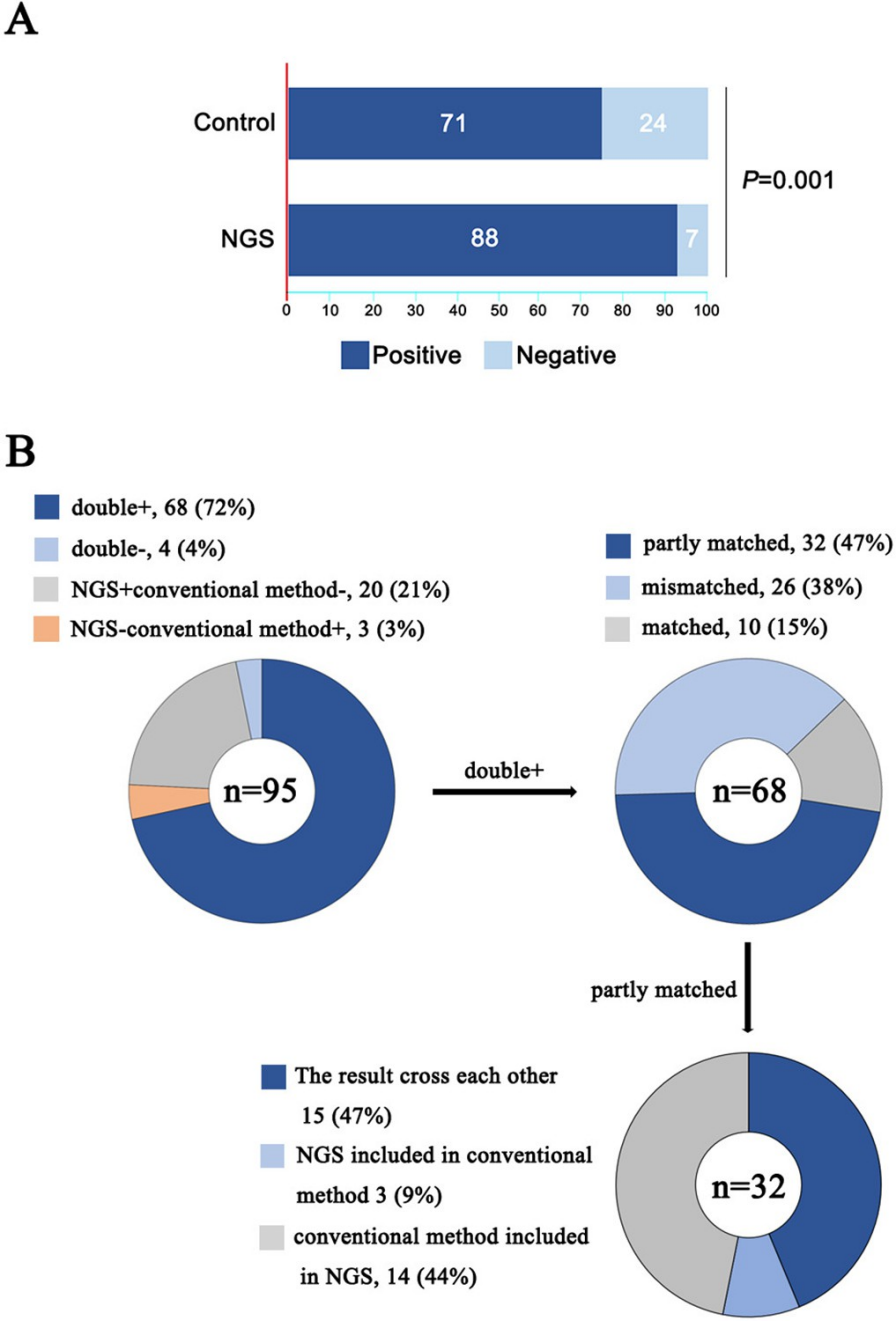
Comparison of diagnostic performance between NGS and conventional pathogen detection methods. (A) comparison of positivity rate between NGS and conventional group. (B) consistency of the NGS and conventional pathogen detection methods.

The diagnostic positive rate of NGS group is significantly higher than that of conventional groups (92.6% vs. 74.7%, *P* = 0.001) (**Fig 3A)**. We analyzed the concordance between NGS and conventional method in SCAP patients with empiric antimicrobial therapy failure in NGS group. In our results, NGS and conventional method detection were both positive in 68 of 95 (72%) cases and were both negative in 4 of 95 (4%) cases. 20 cases were positive by only NGS (21%), and 3 were positive by only culture (3%). In the double-positive group, the test results were considered to be perfectly matched when the pathogens identified by both methods were identical. The result was partly matched when pathogens identified by both methods were partially congruent. The result was mismatched when the pathogens identified by both methods were completely different. Our results indicated that NGS and conventional method were perfectly matched in 10 of 68 cases and totally mismatched in 26 of 68 cases. The remaining 32 cases were found to be “partly matched”, indicative of at least 1 overlapping pathogen when polymicrobial results were observed. In the partly matched ones, 14 cases were conventional method result included in NGS result, while 3cases were NGS result included in conventional method result, 47% were the results cross each other (**Fig 3B**).

### Pathogen spectrum of NGS and conventional methods

**Fig 4:**
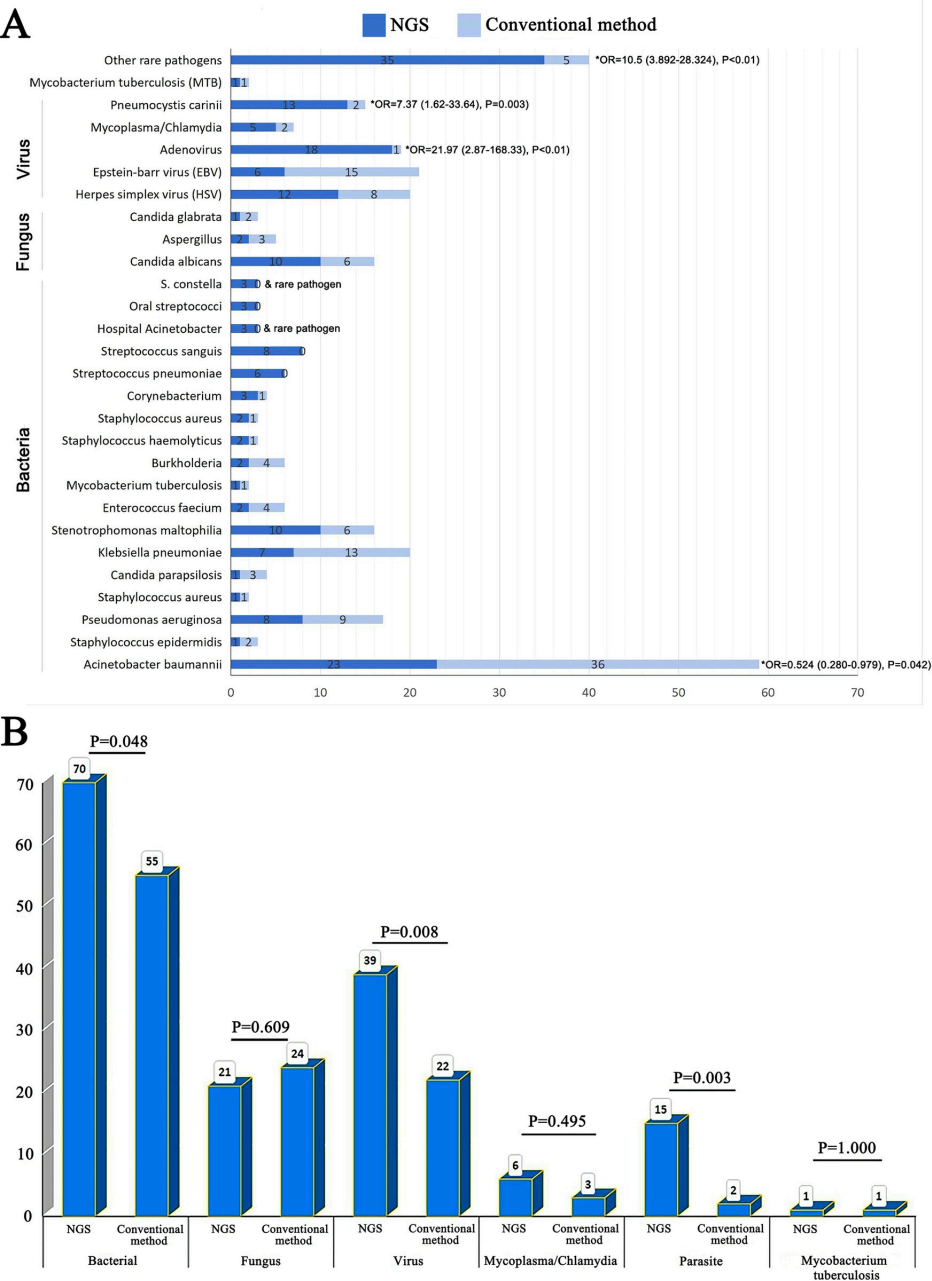
Pathogen spectrum of NGS and conventional methods. (A) overall pathogens-detection yield in the study cohort and the contribution of NGS and conventional methods to the determination of etiology. (B) number of positive samples of bacteria, fungi, viruses, Mycoplasma/chlamydia, parasites, and Mycobacterium tuberculosis detected by NGS and conventional methods.

The corresponding frequencies of different pathogens are plotted in histograms (**Fig 4A**). Rare pathogens demonstrated a superior positivity rate in NGS compared to that in conventional detection methods [OR = 10.5 (95%CI, 3.892–28.324), P<0.01]. Moreover, compared to conventional methods, the NGS detection method had an obvious advantage in detecting Pneumocystis [OR = 7.37 (95%CI, 1.62–33.64), P = 0.003] and adenovirus [OR = 21.974(95%CI, 2.87–168.33), P<0.01], suggesting an important guiding role in anti-infection treatment of severe pneumonia. *Acinetobacter baumannii* had a higher positivity rate in the conventional detection methods than in NGS [OR = 0.524 (95%CI, 0.280–0.979), P = 0.042]. In our study, the positive rate of NGS for bacteria (70/95 vs 55/95, P = 0.048), viruses (39/95 vs 22/95, P = 0.008), and parasites (15/95 vs 22/95, P = 0.003) was significantly higher than that of conventional methods. However, there were no differences in fungi (21/95 vs 24/95, P = 0.609), *Mycobacterium tuberculosis* (MTB) (1/95 vs 1/95, P = 1.00), and Mycoplasma/chlamydia (6/95 vs 3/95, P = 0.495) (**Fig 4B**).

### Mixed infections and co-pathogens

**Fig 5:**
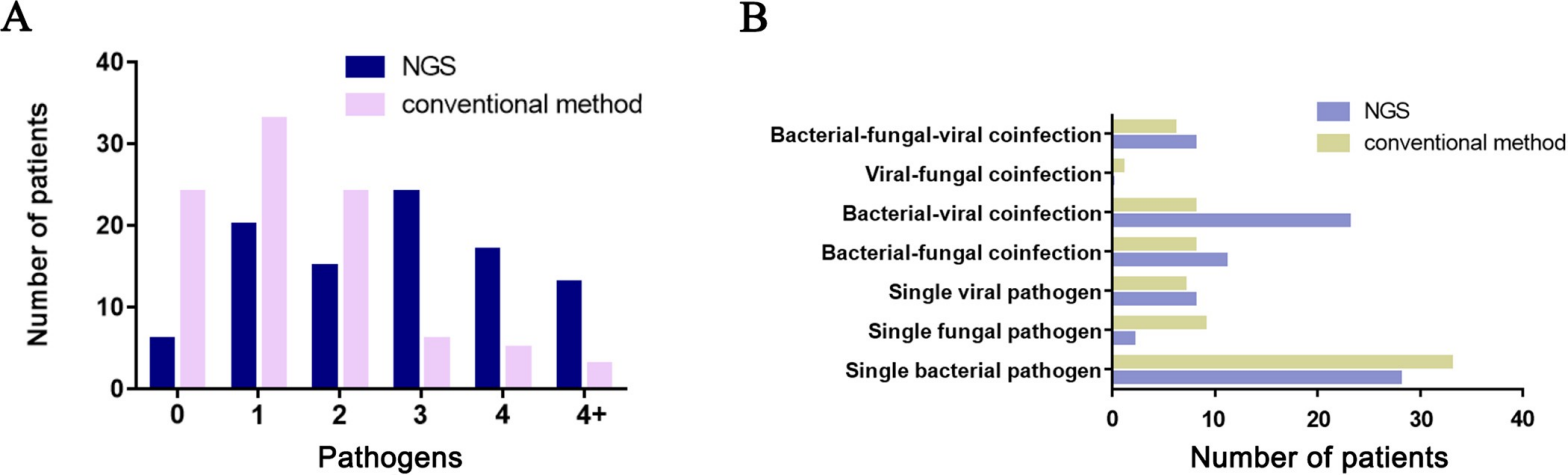
Mixed infection and co-pathogens identified by NGS and conventional methods. (A) number of SCAP patients with mixed infections. (B) number patients with co-pathogens for various microbes.

Numbers of patients with mixed infections detected by NGS and conventional methods are summarized in **Fig 5**. Compared with conventional methods, NGS can identify more pathogens. NGS identified only one potential pathogen in 20 of the 89 patients with empiric antimicrobial therapy failure, two potential pathogens in 15 patients, three pathogens in 24 patients, four pathogens in 17 patients, and more than four pathogens in 13 patients (**Fig 5A**). Viruses and bacteria were the most common co-pathogens observed in SCAP patients with empiric antimicrobial therapy failure (**Fig 5B**).

### Impact of NGS on antimicrobial treatment of SCAP patients with empiric antimicrobial therapy failure

Records of antimicrobial treatment during hospitalization were retrieved from 82 SCAP patients with empiric antimicrobial therapy failure. Based on the results of NGS testing, the antimicrobial treatment was modified in 60 (73.2%) of patients. 26 (31.7%) of patients had one or two antimicrobial agents removed. 13 (15.9%) patients had the antimicrobial spectrum reduced. The number of antimicrobial agents was escalated in 18 (22.0%) patients. Most of the escalation events were related to adding antimicrobial agents against specific pathogens (**Table 3**).

**Table 3:** Impact of NGS on antimicrobial treatment on SCAP patients with empiric antimicrobial therapy failure.

Modifications	Antimicrobial agents	*N* (%)
Remove 1 agent		22 (26.8%)
	TMP-SMZ	4
	MEM	5
	MXF	7
	CAS	6
Remove 2 agent		4 (4.9%)
	TMP-SMZ+CAS	4
Reduce spectrum of agent		13 (15.9%)
Add 1 agent		18 (22.0%)
	VAN	2
	TMP-SMZ	5
	CAS	6
	MEM	3
	Oseltamivir	2
Add 2 agent		3 (3.7%)
	TMP-SMZ+CAS	3
No change		22 (26.8)

TMP-SMZ: sulfamethoxazole and trimethoprim; MEM: meropenem; MXF: moxifloxacin; CAS: caspofungin; VAN: vancomycin; Remove 1 (or 2) agent, the number of antimicrobial agent types reduced by 1 (or 2) after the report of mNGS results; Add 1 (or 2) agent, the number of antimicrobial agent types reduced by 1 (or 2) after the report of mNGS results.

## Discussion

We retrospectively analyzed the difference in efficacy between NGS-guided and conventional pathogen detection-guided antimicrobial treatment. Firstly, NGS can generally detect pathogens in 9–48 hours, which is a shorter time than that required for conventional detection methods (average of 3–5 days) [20–23]. This discrepancy may be one of the reasons for the reduction of mortality and improvement of severe pneumonia after NGS was used to guide treatment. Secondly, the overall pathogen-detection yield of NGS was significantly higher than that of conventional methods (92.6% vs. 74.7%, P = 0.001), which may be attributed to NGS achieving unbiased detection of pathogens by mapping the sequencing reads of a patient sample to the known reference bacteria and viruses database. From the distribution of pathogens, NGS has an obvious advantage in detecting bacteria, viruses, parasites. In addition, NGS was advantageous to identify pneumocystis, adenoviruses, and other rare pathogens. This conclusion was consistent with a previous study in that the positive of NGS for non-HIV-infected Pneumocystis jirovecii Pneumonia patients is significantly higher than that of conventional methods [18]. Furthermore, we analyzed the consistency between NGS and conventional method. The NGS and conventional detection methods were both positive in 68 of 95 (68%) cases, and 32 of 68 cases were found to be “partly matched”. In the partly matched ones, 14 cases were conventional method result included in NGS result, while 3 cases were NGS result included in conventional method result. This indicates that NGS has more advantages in detecting pathogens, in most cases, the pathogenic results of NGS can cover the results of conventional methods.

In this study, SCAP patients who are failed in initial empiric antimicrobial therapy usually have mixed infections. Only 21% of SCAP patients infected with a single pathogen. This indicates that a single antimicrobial spectrum treatment is difficult to achieve ideal efficacy. Our results show that NGS can identify more mixed infections and co-pathogens than conventional methods, providing clinicians with more valuable information to adjust antimicrobial treatment. In SCAP patients with mixed infections, the most common combinations were bacterial-viral coinfection, which is consistent with previous research [15]. This indicates that clinicians should pay more attention to the presence of mixed infections when diagnosing SCAP patients as single bacterial or viral infections.

Furthermore, we compared the change of clinical indicators from admission to discharge and main outcomes between the NGS group and the conventional group. The results showed that NGS group had more antimicrobial treatment adjustments (73.2% vs. 54.9%, *P* = 0.015). In the NGS group, 27.4% of patients removed 1–2 antimicrobial agents, and 22.1% patients added 1–2 antimicrobial agents, which indicated that more microbes detected by NGS may not necessarily the cause of the disease. Clinicians need to further distinguish between background, colonization pathogens, pollution and causative pathogens. Due to faster and more effective adjustment of the anti-infection regimen, it was found that the changes in ESR, CRP, PaO2/FiO2, SOFA score, CURB-65 score and APACHE II score from admission to discharge were significantly higher than that in conventional group. Previous research has shown that high APACHE II score, and high SOFA score were risk factors for SCAP death [24]. This indicates that the NGS test results have a positive effect on clinical medication guidance. More importantly, the mortality of NGS group was lower than that of conventional group (28.0% vs. 43.9%, P = 0.034). Therefore, early and accurate targeted antimicrobial therapy is important to improve patient prognosis.

Although NGS can help clinicians quickly diagnose unexplained respiratory diseases, there are also many limitations. (1) The range of pathogens detected by different techniques and clinical metagenomics platforms are different, and there is currently no unified standard for testing. (2) False positive or false negative may still occur in NGS testing, which may lead to overdiagnosis or overtreatment [25]. (3) According to the current technological workflow, most laboratories require 9–48 hours from samples collection to report results, as the analysis involves multiple complex steps of laboratory processing and bioinformatics analysis. (4) The NGS test performed in this study only detected the pathogen, but did not detect drug resistance, which reduces the accuracy of the medication decision to some extent. It is necessary to refer to both NGS and conventional pathogen detection results and drug susceptibility testing to achieve the best therapeutic effect [26].

This study showed that NGS can be used to diagnose infections more quickly and more accurately than conventional methods, resulting in a better clinical outcome in SCAP patients with empiric antimicrobial therapy failure. For special populations, such as seniors; those suffering from many underlying diseases, immunosuppression, or repeated hospitalizations; critically ill patients; those for whom conventional methods are repeatedly negative; those with special pathogen infections; and those with poor treatment response, NGS testing should be performed early to ensure timely treatment in patients [27–29].

The main flaw of our research is that the small sample size of the research may lead to insufficient generalization of the conclusions; in addition, although the PS matching was used for controlling confounding factors, it may also lose some meaningful results. In future studies, we hope to conduct prospective studies and increase the sample size to further determine the status of NGS in clinical treatment. Furthermore, we cannot identify the ground true positive cases in each group and calculating the false positive rate and false negative rate. clinical diagnosis is based on the results of pathogen testing, other laboratory tests, clinical manifestations, and reactions to drugs. Most antimicrobial drugs have a broad spectrum of antimicrobial agents. These reasons make it impossible for us to infer the true positive cases. Future studies are required to determine the gold standard for detecting various pathogens to compare the specificity and sensitivity of NGS with conventional methods.

## Conclusion

In summary, timely NGS detection of pathogens can effectively guide the use of antimicrobial drugs in SCAP patients with initial empiric therapy. However, due to the limited time span of the study, further investigations are required.

## Supporting information

S1 Fig Comparison of clinical indicators change between NGS and conventional groups without PS matching. (A) change in CRP, (B) change in ESR, (C) change in PaO2/FiO2, (D) change in SOFA score, (E) change in APACHE II score, and (F) change in CURB-65 score. P-values indicate the differences between patients in the NGS and conventional group. CRP, C-reactive protein; ESR, erythrocyte sedimentation rate; PaO2 /FiO2, ratio of arterial oxygen partial pressure to fractional inspired oxygen; SOFA score, Sequential Organ Failure Assessment score; APACHE II, Acute Physiology and Chronic Health Evaluation.
(TIF)

S1 Table Patient clinical baseline characteristics after PS matching. (PDF)

S2 Table Clinical Outcomes of the NGS group and control group without PS matching. (PDF)

S3 Table Change of clinical indicators from admission to discharge after PS. (PDF)
