# Supplementary figures and images for: Next-generation sequencing guides the treatment of severe community-acquired pneumonia with empiric antimicrobial therapy failure: A propensity-score-matched study
Source: PLoS Negl Trop Dis. 2024 Dec 2;18(12):e0012701. doi: 10.1371/journal.pntd.0012701 (PMC11637351; doi:10.1371/journal.pntd.0012701)

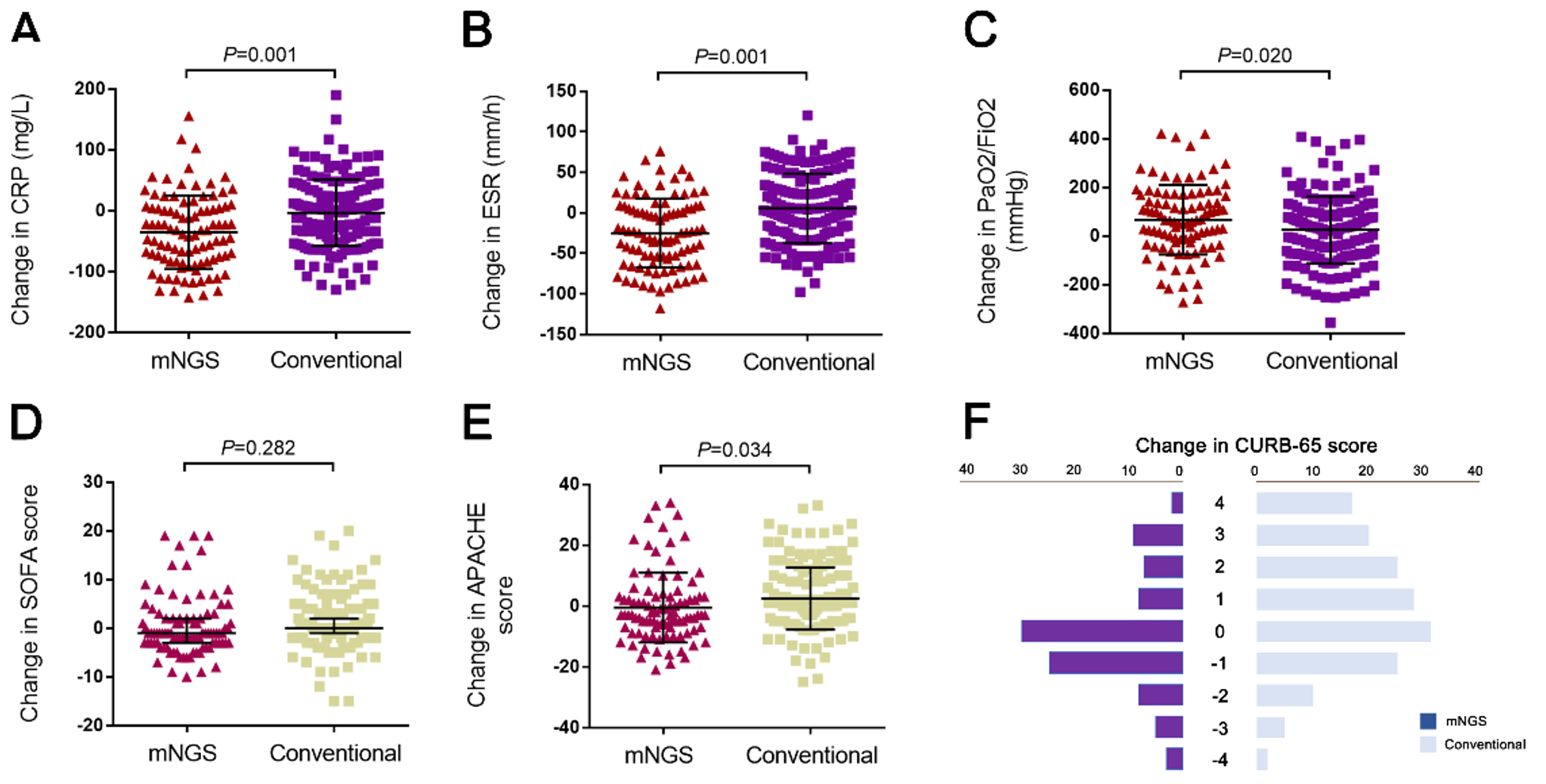

Supplement: S1 Fig — (A) change in CRP, (B) change in ESR, (C) change in PaO2/FiO2, (D) change in SOFA score, (E) change in APACHE II score, and (F) change in CURB-65 score. P-values indicate the differences between patients in the NGS and conventional group. CRP, C-reactive protein; ESR, erythrocyte sedimentation rate; PaO2 /FiO2, ratio of arterial oxygen partial pressure to fractional inspired oxygen; SOFA score, Sequential Organ Failure Assessment score; APACHE II, Acute Physiology and Chronic Health Evaluation. (TIF) [file pntd.0012701.s001.tif]
