# Supplementary material for: Next-generation sequencing guides the treatment of severe community-acquired pneumonia with empiric antimicrobial therapy failure: A propensity-score-matched study
Source: PLoS Negl Trop Dis. 2024 Dec 2;18(12):e0012701. doi: 10.1371/journal.pntd.0012701 (PMC11637351; doi:10.1371/journal.pntd.0012701)
Supplement: S1 Table — (PDF) [file pntd.0012701.s002.pdf]

S1 Table. Patient clinical baseline characteristics without PS matching.

Parameter	NGS group (n=95)	Conventional group (n=163)	P value
Age, year	54.7 (15.9)	63.2 (13.7)	0.12
Sex, male/female	73/22	117/46	0.37
**Comorbidities, n (%)**	-	-	-
chronic heart failure	22 (23.2)	54 (33.1)	0.09
diabetes	13 (13.7)	39 (23.9)	0.05
chronic liver disease	22 (23.2)	49 (30.1)	0.23
COPD	14 (14.7)	31 (19.0)	0.38
cerebrovascular disease	34 (35.8)	67 (41.1)	0.40
tumor	11 (11.6)	28 (17.2)	0.23
**Complications, n (%)**	-	-	-
sepsis	25 (26.3)	44 (27.0)	0.91
MODS	18 (18.9)	24 (14.7)	0.38
ARDS	21 (22.1)	24 (14.7)	0.13
PCT(μg/L)	0.8 (0.2-3.8)	1.1 (0.3-3.7)	0.67
D-dimer	1.5 (0.7-3.1)	1.4 (0.7-3.5)	0.88
PaO2/ FiO2(mmHg)	181.5 (99.8)	204.7 (150.6)	0.30
SOFA score	5 (3-7)	4 (3-7)	0.65
APACHE II score	15.6 (6.9)	18.0 (6.6)	0.74
NE%	81.7 (15.4)	83.2 (15.3)	0.91
PLT (10^9/L)	186.2 (131.3)	224.1 (130.2)	0.40
ESR (mm/h)	72.0 (32.0-96.0)	71.5 (37.0-109.0)	0.61
CRP (mg/L)	125.1 (88.3)	114.4 (77.0)	0.39

COPD: chronic obstructive pulmonary disease; MODS: multiple organ dysfunction syndrome; ARDS: acute respiratory distress syndrome; PCT: procalcitonin; PaO2 /FiO2: ratio of arterial oxygen partial pressure to fractional inspired oxygen; SOFA score: Sequential Organ Failure Assessment score; APACHE II: Acute Physiology and Chronic Health Evaluation; NE%: percentage of neutrophils; PLT: platelets; ESR: erythrocyte sedimentation rate; CRP: C-reactive protein; SD: standard deviation; IQR: interquartile range.
