# Supplementary material for: Next-generation sequencing guides the treatment of severe community-acquired pneumonia with empiric antimicrobial therapy failure: A propensity-score-matched study
Source: PLoS Negl Trop Dis. 2024 Dec 2;18(12):e0012701. doi: 10.1371/journal.pntd.0012701 (PMC11637351; doi:10.1371/journal.pntd.0012701)
Supplement: S2 Table — (PDF) [file pntd.0012701.s003.pdf]

S2 Table. Clinical Outcomes of the NGS group and control group without PS matching

Parameter	NGS group (n=95)	Conventional group (n=163)	P Value
**Primary outcomes**			
Mortality during hospitalization	26/95 (27.3)	69/163 (42.3)	0.016
Improvement rate during hospitalization	61/95 (64.2)	62/163 (38.0)	0.001
**Second outcomes**			
**Duration of mechanical ventilation**			
Mechanical ventilation time, Median	137 (32-250)	184 (63-350)	0.032
Ventilator-free hours, Median	208 (8-320)	134 (5-362)	0.210
**Length of hospital stay(day)**			
ICU hospitalization time	10 (6-18)	11 (3-24)	0.609
Total length of hospital stay	17 (10-26)	16 (7-30)	0.595
**Expenses of antibiotic (RMB, Yuan)**			
Antibiotic cost	21262.8 (27098.6)	252918.6 (25097.2)	0.034
Antibiotic cost/day	1027.4 (672.4- 2311.4)	1397.3 (997.5- 2507.1)	0.041

ICU: intensive care units
