# Supplementary material for: Next-generation sequencing guides the treatment of severe community-acquired pneumonia with empiric antimicrobial therapy failure: A propensity-score-matched study
Source: PLoS Negl Trop Dis. 2024 Dec 2;18(12):e0012701. doi: 10.1371/journal.pntd.0012701 (PMC11637351; doi:10.1371/journal.pntd.0012701)
Supplement: S3 Table — (PDF) [file pntd.0012701.s004.pdf]

S3 Table. Change of clinical indicators from admission to discharge after PS

Parameter	NGS group (n=82)	Control group (n=82)	P Value
Change in NE%	-5.5 (-17.6-3.2)	-1.5 (-11.1-4.6)	0.200
Change in L%	4.7 (-1.0-11.6)	-1.5 (-11.1-4.6)	0.200
Change in PLT (10^9/L),	17.0 (-80.3-122.3)	16.0 (-77.8-131.5)	0.960
Change in CRP (mg/L)	-63.0 (-113.2-0.0)	3.0 (-60.3-39.4)	0.006
Change in PCT (mg/L)	-0.2 (-3.1-1.1)	-0.0 (-1.2-2.2)	0.450
Change in ESR (mm/h)	-31.0 (-55.0- -2.0)	0.5 (-17.2-30.0)	0.006
Change in D-dimer (mg/L)	0.1 (-1.6-2.2)	0.0 (-1.2-1.3)	0.630
Change in AST (U/L)	-5.6 (-10.7- -3.5)	-4.6 (-7.3- -2.2)	0.570
Change in ALT (U/L)	-11.2 (-19.5- -5.6)	-9.8 (-17.4- -5.0)	0.644
Change in urea (mmol/L)	-3.3 (-8.5-1.0)	-2.7 (-7.9-1.7)	0.763
Change in creatinine (μ mol/L)	-8.2 (-17.1- -2.3)	-7.6 (-16.8- -1.1)	0.707
Change in NT-proBNP (pg/ml)	4.3 (-1839.0-2748.0)	898.0 (-404.0-6172.0)	0.005
Change in PaO2/ FiO2 (mmHg),	66.9 (-43.1-170.3)	6.5 (-82.4-113.1)	0.043
Change in SOFA score	-1 (-3-2)	1 (-2-6.5)	0.005
SOFA score improved	53/81 (65.4)	31/77 (40.3)	0.027
Change in CURB-65 score	0 (-1-1)	0.5 (-1-2)	0.006
CURB-65 score improved	34/82 (41.5)	21/82 (25.6)	0.002
Change in APACHE II	-3 (-8-3)	0 (-5-12)	0.011
APACHE II improved	50/81 (61.7)	34/77 (44.2)	0.027

NE%: percentage of neutrophils; L%: percentage of lymphocyte; PLT: platelets; CRP: C-reactive protein; PCT: procalcitonin; ESR: erythrocyte sedimentation rate; AST: aspartate aminotransferase; ALT: alanine aminotransferase; NT-proBNP: amino-terminal pro-brain natriuretic peptide; NT-proBNP: N-terminal pro B-type natriuretic peptide; PaO2 /FiO2: ratio of arterial oxygen partial pressure to fractional inspired oxygen; SOFA score: Sequential Organ Failure Assessment score; APACHE II: Acute Physiology and Chronic Health Evaluation.
